# Optimization of Capacitive Acoustic Resonant Sensor Using Numerical Simulation and Design of Experiment

**DOI:** 10.3390/s150408945

**Published:** 2015-04-16

**Authors:** Rubaiyet Iftekharul Haque, Christophe Loussert, Michelle Sergent, Patrick Benaben, Xavier Boddaert

**Affiliations:** 1 Centre Microélectronique de Provence, Ecole des Mines de Saint-Etienne, Gardanne 13541, France; E-Mail: benaben@emse.fr; 2 TAGSYS RFID, 13600 La Ciotat, France; E-Mail: christophe.loussert@tagsysrfid.com; 3 Aix-Marseille Université, LISA EA 4672, 13397 Marseille Cedex 20, France; E-Mail: michelle.sergent@univ-amu.fr

**Keywords:** acoustic sensor, resonance, numerical simulation, design of experiments, response surface method, optimization

## Abstract

Optimization of the acoustic resonant sensor requires a clear understanding of how the output responses of the sensor are affected by the variation of different factors. During this work, output responses of a capacitive acoustic transducer, such as membrane displacement, quality factor, and capacitance variation, are considered to evaluate the sensor design. The six device parameters taken into consideration are membrane radius, backplate radius, cavity height, air gap, membrane tension, and membrane thickness. The effects of factors on the output responses of the transducer are investigated using an integrated methodology that combines numerical simulation and design of experiments (DOE). A series of numerical experiments are conducted to obtain output responses for different combinations of device parameters using finite element methods (FEM). Response surface method is used to identify the significant factors and to develop the empirical models for the output responses. Finally, these results are utilized to calculate the optimum device parameters using multi-criteria optimization with desirability function. Thereafter, the validating experiments are designed and deployed using the numerical simulation to crosscheck the responses.

## 1. Introduction

For many years, acoustic sensors have been used in many civilian and military applications, such as in cellular phones, hearing aids, and computers, in addition to high quality studio microphones for sound recording [1], sonar for underwater objects detection [2], and in the acoustic sensor systems for target acquisition and surveillance purposes [3] *etc.* An acoustic transducer provides analog output that is proportional to the variation of acoustic pressure acting upon a flexible diaphragm. Most familiar examples of acoustic sensors are the microphone, earphone *etc.* There are different types of acoustic sensors: namely, piezoelectric, piezoresistive, and capacitive [4]. Among them, capacitive acoustic sensors show the highest sensitivity while maintaining low power consumption [5]. Capacitive sensing is independent of the base materials and relies on the variation of the capacitance when geometry of a capacitor is changing. Furthermore, capacitive acoustic sensor can be used as both an active and passive sensing device.

A capacitive acoustic transducer is an electromechanical-acoustic system. It usually consists of a fixed backplate electrode and a flexible diaphragm that acts as a second electrode, separated by a dielectric material, such as air, to form a parallel plate capacitor. The deflection of the diaphragm occurs due to incident acoustic pressure, thereby providing capacitance variation in response to the change in air gap. In general, capacitive acoustic transducer suffers from over-damping, as a thin layer of air is trapped in between the electrodes; therefore, capacitive acoustic transducers are usually designed and fabricated with porous membranes or/and perforated backplates to reduce the damping effect.

To date, many capacitive acoustic sensors have been developed, and some of them are commercially available. However, its design varies based on the application domains, and the majority of these are targeted for audio applications with nearly uniform sensitivity over a relatively wide range of frequencies in the human hearing range, 20 Hz–20 kHz [4,5,6,7,8,9,10].

Recently, a new simplified design concept has been proposed to fabricate a capacitive acoustic transducer, which consists of a central cylindrical rigid backing electrode of small radius surrounded by a flat annular cavity below a vibrating membrane clamped at its periphery separated by an air gap, which provides good sensitivity and a large frequency bandwidth [9,10]. Honzik *et al.* [9] have reported that this design leads to a higher sensitivity, as well as a larger frequency bandwidth.

A capacitive acoustic sensor, similar to that of a condenser microphone, can also be used as an acoustic resonant sensor by modifying different parameters related to the device fabrication. The characteristics of the damping material and other geometric parameters determine the transducer bandwidth. Generally, transducers respond to incident acoustic pressure over the entire range of relevant frequencies, whereas resonant transducers provide higher sensitivity at their natural frequencies.

**Figure 1:**
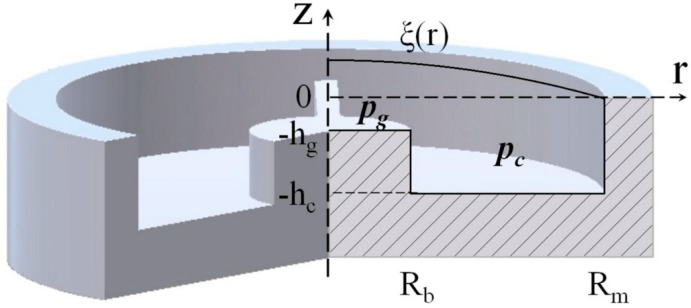
Schematic diagram of acoustic transducer.

The design presented in Figure 1 can be a potential candidate to fabricate a capacitive acoustic resonant sensor with good selectivity at a certain frequency. During this work, we investigate the possibilities to develop the acoustic resonator based on this simplified design concept. To fulfill the specific system requirement, a capacitive resonant sensor with strong sensitivity at specified frequency with narrow bandwidth is desired. To do so, one needs to optimize structural parameters, such as membrane radius, backplate radius, air gap, cavity height, membrane thickness, of the design of the acoustic sensor (Figure 1). In addition, the membrane tension and material uses to fabricate the device needed to be optimized, as well.

As a large number of parameters are involved in acoustic sensor optimization, numerical simulation can be a powerful and economical tool for virtual device prototyping. However, the extensive computational effort is involved in numerical simulation and thus it usually takes a substantial amount of time to complete simulation runs of a complex structure. This paper presents a new design scheme for acoustic sensor optimization that combines numerical simulation using the COMSOL Multiphysics software and design of experiments (DOE) approach to optimize the acoustic sensor of the proposed design to obtain the acoustic resonator. DOE helps to develop a plan of experiments that provides a great deal of information about the effect of input parameters on responses. In this scheme, a set of numerical experiments is conducted to generate responses. Thereafter, based on the numerical simulation results, namely, membrane displacement, capacitance variation, quality factor, *etc.*, the response surface method (RSM) is used to derive empirical models for each of the responses, which will later be used for optimization process. The empirical model reduces computational efforts in the acoustic sensor optimization, since they are far less complex than the original finite element model.

In case of a single response characteristic, optimization can simply be obtained by determining the experimental conditions that satisfied the expected response [11]. However, the performance of a capacitive acoustic resonant sensor is often characterized by a group of responses, such as static capacitance, capacitance variation, quality factor, *etc.* If more than one response comes into consideration, it is very difficult to select the optimal setting that can achieve all quality requirements simultaneously.

Vogel *et al.* [12] have applied FEM and a sequential quadratic programming (SQP) method as part of the CAPA optimization module to optimize micromachined capacitive ultrasound transducer array (CMUT), the design of comb structures for use in acceleration sensors, and the optimization of an electrostatic membrane device for an integrated silicone microphone. The SQP method is generally used for a nonlinearly constrained optimization problem that approximately solves a sequence of optimization subproblems, each of which optimizes a quadratic model of the objective subject to a linearization of the constraints. However, it is difficult to implement SQP methods so that exact second derivatives can be used efficiently and reliably [13]. The alternative of this approach, is to make use of a desirability function that transforms an estimated response into a scale-free value, known as global desirability [11,14,15]. With the multi-objective nature of our problem, desirability function is employed during this work to avoid the disadvantages of other methods.

The objectives of this study are to investigate the effect of different parameters of the transducer on the output responses using the numerical analysis and DOE approach, and to optimize device parameters to develop acoustic resonator that provides good sensitivity and selectivity. In this regard, the first part of this paper is devoted to the theoretical analysis to understand the system, and then the construction of the finite element (FE) model of the acoustic sensor (based on the design presented in Figure 1). Thereafter, DOE is introduced to achieve greater information about the effects of different input parameters on output responses with the least possible number of experiments. Finally, multi-criteria optimization is performed to obtain the optimum set of parameters, which is verified using numerical simulation.

## 2. Theoretical Analysis

A capacitive acoustic sensor is an electro-mechanical transducer that transforms the mechanical deformation of the diaphragm in an output signal. The capacitance ($C_{0}$) of a parallel plate capacitor, with a fixed distance between the two electrodes $h_{0}$ and area of overlap of the two electrodes plates $S_{e}$, also known as effective area, is given by

(1) $$C_{0}=\frac{\varepsilon_{0}\varepsilon_{r}S_{e}}{h_{0}}$$

where $\varepsilon_{0}$ represents electric constant ($\varepsilon_{0}=8.854\times 10^{-12}Fm^{-1}$) and $\varepsilon_{r}$ represents the relative static permittivity of the materials between the plates (for a vacuum, $\varepsilon_{r}=1$). When an external DC voltage ($V_{0}$) is applied, an electrostatic force ($F_{es}$) as presented by Equation (2), is created across the electrodes and induces a membrane deformation.

(2) $$F_{es}=\frac{Q^{2}}{2\varepsilon_{0}S_{e}}=\frac{\varepsilon_{0}S_{e}}{2h_{0}^{2}}V_{0}^{2}$$

Thus the air gap ($h_{g}$) becomes ($h_{0}+{\langle \xi \rangle }_{es}$), where ${\langle \xi \rangle }_{es}$ represents the quiescent average deformation of membrane due to the electrostatic forces of the pre-polarization of the transducer. Therefore, the static capacitance ($C_{i}$) of the acoustic sensor can be expressed as,

(3) $$C_{i}=\frac{\varepsilon_{0}\varepsilon_{r}S_{e}}{h_{0}+{\langle \xi \rangle }_{es}}=\frac{\varepsilon_{0}\varepsilon_{r}S_{e}}{h_{g}}$$

The air gap of the transducer varies due to membrane deformation. If ${\langle \xi \rangle }_{Se}$ represents the average small-signal deformation; the varied distance between the back electrode and the membrane becomes $h_{g}+{\langle \xi \rangle }_{Se}$. Thus, the output capacitance ($C_{n}$) due to incident pressure can be expressed as follows,

(4) $$C_{n}=\frac{\varepsilon_{0}\varepsilon_{r}S_{e}}{h_{g}+{\langle \xi \rangle }_{Se}}=\frac{\varepsilon_{0}\varepsilon_{r}S_{e}}{h_{g}}\left(1-\frac{{\langle \xi \rangle }_{Se}}{h_{g}}\right)=C_{i}\left(1-\frac{{\langle \xi \rangle }_{Se}}{h_{g}}\right)$$

where the expression has been expanded to the first order (Taylor series). Therefore, the capacitance variation (ΔC) can be obtained by subtracting Equation (4) from Equation (3),

(5) $$\Delta C=\left|C_{n}-C_{i}\right|=C_{i}\frac{\left|{\langle \xi \rangle }_{Se}\right|}{h_{g}}$$

On the other hand, the total voltage (V) across the capacitor is the sum of the quiescent polarization voltage ($V_{0}$) and the small-signal output voltage ($V_{out}$). The charge (Q) in the capacitor can be expressed as,

(6) Q=CV

Its differentiation is given as:

(7) dQ=CdV+VdC

We assume that the system has a constant total charge, $Q=\sum_{i}q_{i}=\;const.$, thus dQ=0. The inclusion of this assumption in Equation (7) gives

(8) $$dV=-\frac{dC}{C}V$$

Assuming $C=C_{n}$, dC≈ΔC, $dV\approx V_{out}$, $V\approx V_{0}$, and introducing them into Equation (8):

(9) $$V_{out}=V_{0}\frac{C_{i}\frac{{\langle \xi \rangle }_{Se}}{h_{g}}}{C_{i}\left(1-\frac{{\langle \xi \rangle }_{Se}}{h_{g}}\right)}=V_{0}\frac{{\langle \xi \rangle }_{Se}}{h_{g}}\left(1+\frac{{\langle \xi \rangle }_{Se}}{h_{g}}\right)\approx V_{0}\frac{{\langle \xi \rangle }_{Se}}{h_{g}}$$

The expression has been expanded to the first order (Taylor series), and the higher order term is negligible and thus removed. Based on the analysis, it has been observed that the capacitance variation as well as output voltage of the acoustic sensor mainly depends on the membrane displacement. Therefore, to improve the sensitivity of the sensor, we have to design the sensor which will provide high membrane displacement.

### 2.1. Equations Governing the Membrane Displacement

The equation governing the vibration of the thin circular membrane of thickness $t_{m}$, radius $R_{m}$, and density $\rho_{m}$ under constant radial force per unit length ($T_{m}$) acting on its edge, driven by uniform harmonic incident acoustic pressure $p_{i}$ over the membrane surface, loaded by the pressure field p(r), also known as reaction pressure at the membrane surface, can be expressed as [9,10,16,17]:

(10) $$T_{m}\left(\Delta_{r}+K^{2}\right)\xi \left(r\right)=p_{i}-p\left(r\right),\;\;\;\;\;0<r<R_{m}$$

Here, ξ(r) being the vertical membrane displacement, ${∆}_{r}$ (equals to $\nabla_{r}^{2}$) represents the Laplace operator, and $K^{2}$ defines the wavenumber of the free flexural vibration of the membrane,

(11) $$K^{2}=\frac{\omega^{2}\rho_{ms}}{T_{m}}=\frac{\omega^{2}}{c^{2}};\;c=\sqrt{\frac{T_{m}}{\rho_{ms}}}\;\mathrm{and}\;\rho_{ms}=t_{m}\rho_{m}$$

where, c denotes the speed of sound in the membrane, $\rho_{ms}$ being the surface density or mass per unit area of the membrane and ω is the angular frequency. The membrane is supported on a rigid circular frame at its periphery $r=R_{m}$ (Dirichlet boundary condition), therefore

(12) $$\xi (R_{m})=0$$

The reaction pressure p(r), loading the diaphragm, is due to the underlying air layer squeezed in the air gap and in the annular cavity under the membrane, where

(13) $$p\left(r\right)=\{\begin{array}{cc} p_{g}\left(r\right)\;\;\;\;\;\;\;\; & r\in \left(0,R_{e}\right)\\ p_{c}=const & r\in \left(R_{e},R_{m}\right) \end{array}$$

Here, $p_{g}(r)$ and $p_{c}(r)$ represent the pressure in the air gap and the pressure in the cavity volume which is assumed to be quasi-uniform, respectively, and $R_{e}$ represents the effective radius and is equal to the radii of the backplate electrode ($R_{b}$).

The incident acoustic signal (with the time factor given by $e^{j\omega t}$) triggers the membrane displacement ξ(r), which is assumed to be small and harmonic ($\xi (r)e^{j\omega t}$). The membrane displacement gives rise to the motion of the air in the domain below the circular membrane, composed of the air gap and annular cavity. It is assumed that the pressure variation in air gap and cavity region are constant throughout the thickness of the fluid film; it depends only on the tangent coordinate r. As the pressure variation does not depend on the z-coordinate, the z-component of the particle velocity (ν) can be neglected. On the other hand, the temperature variation (τ), depends on both coordinates r and z, which is approximately proportional to the pressure variation outside the boundary layers. The temperature variation vanishes at the interfaces between the fluid layer and the membrane z=0, and between the fluid layer and the backing electrode $z=-h_{g}$. Thus the boundary conditions associated with the system are,

(14) $$v_{r(g,c)}(r,0)=v_{r(g,c)}(r,-h_{g,c})=0\;\mathrm{and}\;\tau_{g,c}(r,0)=\tau_{g,c}(r,-h_{g,c})=0$$

The solution of the mean displacement of the circular membrane over the backplate electrode driven by the constant incident pressure $p_{i}$ due to the sound field can be expressed as follows [9,10]:

(15) $${\langle \xi \rangle }_{Se}=\frac{1}{S_{e}}\iint_{S_{e}}\xi (r)dS_{e}=\frac{2}{\sqrt{\pi }R_{e}R_{m}}\sum_{n}\xi_{n}\frac{J_{1}(K_{n}R_{e})}{K_{n}J_{1}(K_{n}R_{m})}$$

### 2.2. Pressure Sensitivity

The sensitivity level (L) of the acoustic sensor for the given polarization voltage $V_{0}$, represents the relation between the input pressure and the output voltage, and can be expressed as follows:

(16) $$L=20\log\left[\left|\frac{V_{out}}{p_{i}}\right|\right]=20\log\left[\left|\frac{V_{0}{\langle \xi \rangle }_{Se}}{p_{i}h_{g}}\right|\right]$$

### 2.3. Resonance Frequency

The selectivity of the acoustic resonant sensor depends on its natural frequency or resonance frequency. At resonance frequency, all parts of the membrane vibrate sinusoidally with the same frequency and with a fixed phase relation, which provides maximum displacement of membrane and is known as normal mode of vibration. Resonance frequencies of the membrane in vacuum are solely determined by its physical dimensions and mechanical constants: namely, Young’s modulus, density of the membrane materials, size of membrane, and boundary conditions. As the maximum membrane displacement occurs at resonance frequency, it leads to the maximum sensitivity for the capacitive acoustic sensor. The natural frequencies of the pre-tensioned circular vibrating membrane in vacuum is given by [18,19],

(17) $$f_{ij}=\frac{k_{ij}}{2\pi R_{m}}\sqrt{\frac{T_{m}}{t_{m}\rho_{m}}}=\frac{k_{ij}}{2\pi R_{m}}\sqrt{\frac{T_{m}}{\rho_{ms}}}$$

The values $k_{ij}$ are derived from the roots of the Bessel functions of the first kind. The natural frequencies of vibration and mode shapes are identified by two integers (i, j) that characterize the mode shape. The index i=1, 2, 3, ... corresponds to the number of circumferential lines (with r=const.) on the membrane that have zero displacement, while j= 0, 1, 2, … corresponds to the number of diametral lines (with θ=const.) that have zero displacement. The values $k_{ij}$ for the first six modes are listed in Table 1.

**Table 1:** Values of $k_{ij}$ derived from the roots of the Bessel functions of the first kind for first six modes.

Mode Number	Factor
1	*k_10_* = 2.4048
2	*k_11_* = 3.8317
3	*k_11_* = 3.8317
4	*k_12_* = 5.1356
5	*k_12_* = 5.1356
6	*k_20_* = 5.5201

However, in the case of a capacitive acoustic resonator, the membrane is usually loaded with an air cavity rather than vibrating in free space [20]. The presence of the cavity generally detunes the membrane resonance [21]. The shifts of first resonance frequency of the system towards the higher frequency than that of the membrane in vacuum occurs due to viscous damping and acoustic stiffness of the cavity.

### 2.4. Quality Factor

The quality factor ($Q_{f}$), which is related to the energy loss of the vibrating diaphragm [22], is characterized by a resonator’s bandwidth relative to its center frequency. Generally, in frequency domain, Q-factor is expressed as,

(18) $$Q_{f}=\frac{f_{r}}{\Delta f}=\frac{\omega_{r}}{\Delta \omega }$$

where, $f_{r}$ is the resonance frequency, ∆f is the half-power bandwidth (*i.e.*, the bandwidth over which the power of the vibration is greater than half the power at the resonance frequency), $\omega_{r}=2\pi f_{r}$ is the angular resonance frequency and ∆ω the angular half power bandwidth.

High $Q_{f}$ value represents low damping, which indicate low rate of energy loss relative to the stored energy of the resonator [23,24]. Q-factor is inversely proportional to the damping coefficient of the oscillating system and define as [23],

(19) $$Q_{f}=2\pi \frac{W}{\Delta W}=\frac{m\omega_{r}}{\gamma }=\frac{K}{\gamma \omega_{r}}$$

where W is the total energy stored in the resonator, ∆W is the sum of the energy loss per cycle, K is the spring constant of the resonator, γ is the coefficient of the damping force, and m is the mass of the oscillator.

Thus, in the case of the acoustic resonator, higher Q-factor represents high selectivity. The Q-factor of the system can be improved by enhancing the total stored energy, while reducing the energy loss per cycle.

## 3. Numerical Simulation

### 3.1. Finite Element Model (FEM)

**Figure 2:**
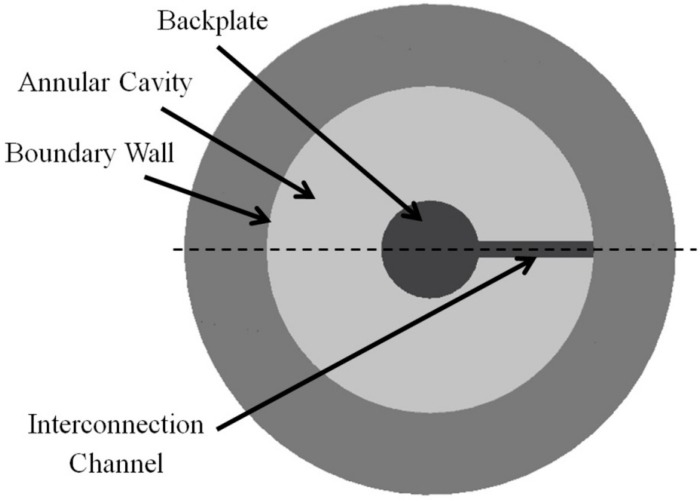
Schematic diagram of top view of the proposed acoustic sensor after removing the diaphragm.

The capacitive acoustic sensor works by transforming the mechanical deformation of the thin membrane (diaphragm), induced by an external incident pressure, into an AC voltage signal. Numerical simulation is performed using finite element method (FEM) not only to understand but also to quantify the effect of different input parameters on the membrane displacement, capacitance variation, Q-factor *etc.* The finite element simulation of the acoustic sensor is a moving boundary problem, in which the computational air domain within the sensor changes continuously, because of membrane vibration under harmonic acoustic wave. Three-dimensional (3D) FEM model is developed using half-slice of the air domain (symmetrical part), as illustrated in Figure 2, to reduce the computational time.

**Figure 3:**
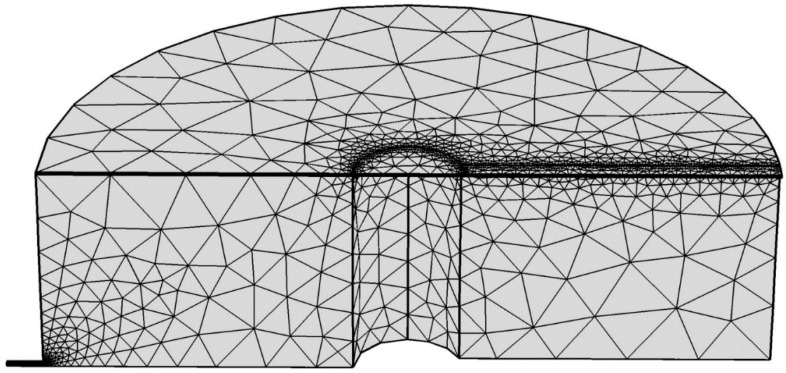
Half-slice of air domain (symmetrical part) of the acoustic sensor with finite element mesh.

Figure 3 illustrates the half-slice of the 3D air domain with finite element mesh, which was solved considering the periodicity and symmetry of the boundary value. The custom mesh is used in such a way that it resolves the acoustic boundary layer for the frequency range of 0 Hz to 250 kHz without mesh regeneration. The physical parameters of the air and the membrane materials are given in Table 2.

**Table 2:** List of parameters of the thermoviscous fluid (air) and material properties of the membrane.

Parameter	Value	Unit
Bulk viscosity (${µ}_{B0}$)	10 × 10^−6^	Pa·s
Gas constant ($R_{s0}$)	281.4	J/(kg·K)
Density of Membrane ($\rho_{m}$)	1390	kg/m^3^
Young’s modulus of membrane ($E_{m}$)	4 × 10^9^	Pa
Poisson’s ratio of membrane ($\upsilon_{m}$)	0.38	-

**Figure 4:**
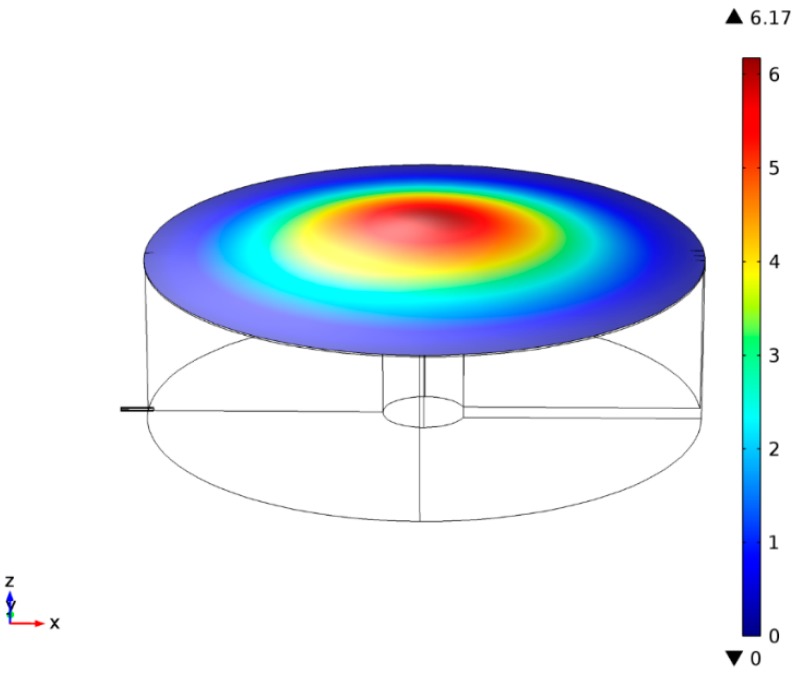
Measured displacement field of the membrane of the acoustic sensor at first resonance frequency ($f_{r}\;$= 16,737 Hz) for $R_{m}\;$= 5 mm, $R_{b}\;$= 0.75 mm, $h_{c}\;$= 3000 µm, $h_{g}$ = 30 µm, $T_{m}\;$= 500 N/m and $t_{m}\;$= 8 µm.

The resulting finite element model with fully coupled thermoacoustic, electrostatic, moving mesh and membrane physics interface was solved using the linear-perturbation solver, PARDISO, in the frequency domain. Solution of the numerical simulation provides the membrane displacement with respect to frequencies. Figure 4 presents the membrane displacement at first resonance frequency ($f_{r}$).

COMSOL Multiphysics software (version 4.4) is used to perform 3D numerical simulation. All the numerical works have been executed on a workstation, DELL PRECISION T7600, having 32 Gigabytes RAM and 16 cores (two 3.1 GHz eight-core Intel Xeon E5-2687W processors).

**Figure 5:**
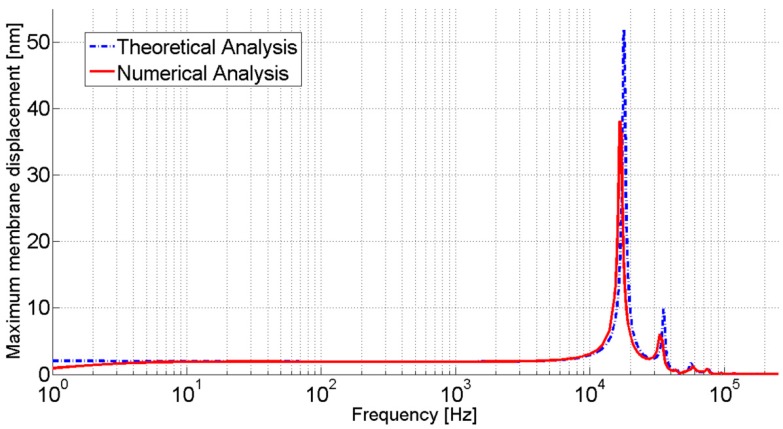
Comparison of the membrane displacement of theoretical and numerical analysis of the acoustic transducer (for $R_{m}$ = 5 mm, $R_{b}$ = 0.75 mm, $h_{c}$ = 3000 µm, $h_{g}$ = 30 µm, $T_{m}\;$= 500 N/m and $t_{m}\;$= 8 µm).

The validation of the numerical model is checked by comparing the maximum membrane displacement of the numerical analysis with that of the theoretical analysis as presented in Equation (15). The results show proximate similarity as depicted in Figure 5. A little shift of the resonance frequency and a slightly smaller magnitude of the membrane displacement are obtained in the FEM results. They are caused by the presence of the interconnection channel to electrically connect the bottom electrode with the outside and venting hole in the geometry as it is in the real device; whereas for simplicity the effect of the interconnecting channel and venting hole are not considered in the theoretical analysis.

### 3.2. Selection of Parameters and Responses

Based on the theoretical analysis and the device structure, several parameters, such as membrane radius ($R_{m}$), bottom electrode radius ($R_{b}$), cavity height ($h_{c}$), air gap ($h_{g}$), membrane tension ($T_{m}$), membrane thickness ($t_{m}$), materials properties (e.g., Young’s modulus, density of the materials and Poisson ratio), and the venting hole geometry, are involved with device performance. During this study, geometry of the venting hole was kept unchanged and the polyethylene terephthalate (PET) thin film was used as a membrane material, whose properties are listed in Table 2. Thus venting-hole geometry and material properties were omitted from the further analysis.

The static capacitance of the system is generally determined by the effective surface area of the electrodes and air gap, whereas the quality factor depends on the damping loss mechanism that is related to the device geometry. On the other hand, the sensitivity of the acoustic sensor, such as capacitance variation, is basically driven by the membrane displacement. Therefore, the first step of this work is identification of major input parameters that strongly influence the membrane displacement and quality factor. In this regard, the classic one-variable-at-a-time method is used, where the effect of individual parameter on the membrane displacement at first resonance frequency was studied for a fixed set of other parameters at some nominal value using numerical simulation. The process is repeated for each of the parameters involved in the study until all the parameters have been studied.

**Figure 6:**
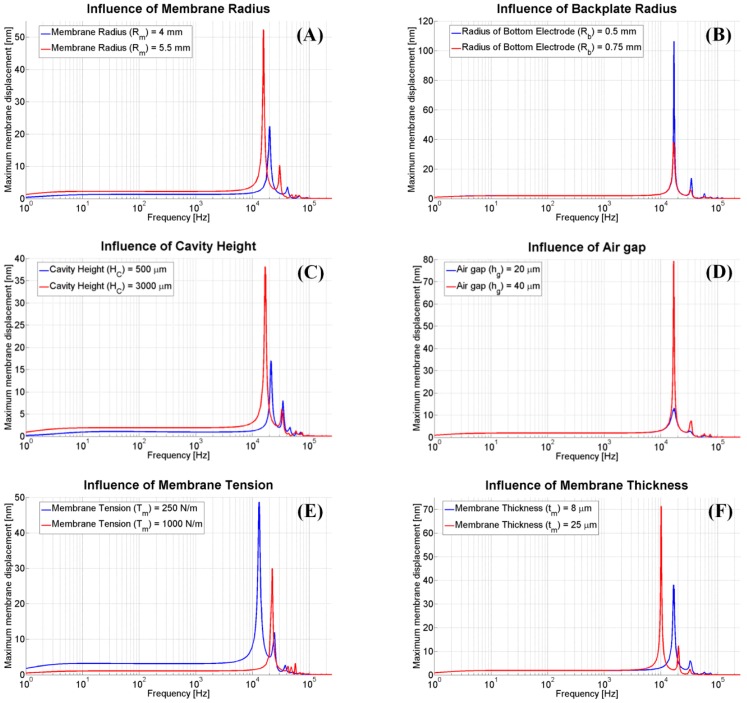
Effect of individual input parameter on the membrane displacement (other parameters kept at constant value: $R_{m}\;$= 5 mm, $R_{b\;}$= 0.75 mm, $h_{c}\;$= 3000 µm, $h_{g}$ = 30 µm, $T_{m}\;$= 500 N/m and $t_{m}\;$= 8 µm).

It has been observed that the increase of the membrane displacement and quality factor, and shift of the resonance frequency are observed for the increasing membrane radius, as shown in Figure 6A. On the other hand, the increase of the bottom electrode radius leads to reduction of membrane displacement and quality factor (Figure 6B). Figure 6C illustrates the effect of cavity height on the membrane displacement. Large cavity height helps to reduce the air damping in the cavity and thus helps to increase the membrane displacement and quality factor. Similarly, increase in air gap provides higher membrane displacement and quality factor (Figure 6D); however, the increase in air gap leads to the lower static capacitance. In addition, as shown in Figure 6E, higher membrane tension reduces the membrane displacement, and also shifts the resonance at a higher frequency. Thickness of the membrane also affects the membrane displacement and the resonance frequency (Figure 6F).

Based on the initial tests, it has been observed that all six input parameters, namely $R_{m}$, $R_{b}$, $h_{c}$, $h_{g}$, $T_{m}$ and $t_{m}$, have some influence on the membrane displacement and Q-factor, and therefore on the output responses. Moreover, to achieve better selectivity, the sensitivity at other natural frequencies than the first resonance frequency has to be reduced. Therefore, in order to study and eventually to optimize the capacitive acoustic resonator to fulfill the requirements, several output responses, specifically static capacitance ($C_{0}$), membrane displacement at first resonance (|˂*ξ_Se_*˃|_*fr1*_), quality factor ($Q_{f}$), capacitance variation (∆C), and membrane displacement at second resonance (|˂*ξ_Se_*˃|*_fr2_*) were studied for each experiment.

However, the one-variable-at-a-time approach cannot predict the interaction between the factors. In addition, this approach is not applicable for multiple response problems, and does not permit the construction of a model for the system [25]. Therefore, the study of the influence of all the input parameters and their interactions, and the optimization of the system requires methodical experimental strategies based on DOE. A good experimental strategy will provide the necessary information to estimate effects of factors and to develop empirical models for each system outputs and to optimize the multiple responses simultaneously to fulfill the objectives.

## 4. Experimental Design

DOE provides a systematic way to study the effects of the input variables of a system or process, also known as factors, on outputs or responses. It is an effective tool for maximizing the amount of information gained from a study while minimizing the number of tests to be performed. In practice, DOE is applicable to both physical processes and numerical simulation models [26,27]; however, unlike physical measurement, numerical experimentation is not subject to noise or uncertainty [28,29].

Compared to other experimental strategies, namely one-variable-at-a-time and sequential simplex, RSM is intended to predict the response with a good quality all over the experimental domain. RSM approach has four basic steps: the data collection according to an experimental design, an empirical model (e.g., polynomial), calculation by least squares regression for each of the responses, generation of response surface contour plots or maps that are examined to the region of the desired response, and finally, the experimental verification of the predicted optimum [25]. DOE coupled with RSM can achieve rapid process development for minimal cost.

The selection of appropriate experiments is very important to build a reliable response surface model and therefore on its precise prediction [30]. According to the postulated model, there are different optimal design of experiments with a guarantee of good prediction in the domain of interest. One of the best known for a second-order model is the class of central composite design (CCD), consisting of a two-level complete or fractional factorial design, an “axial” design, and center points [30].

A series of FEM analyses of an acoustic sensor based on DOE have been performed to investigate the possibility of determining the optimal set of parameters to fabricate a sensor with optimum sensitivity and selectivity. The optimization is carried out to maximize the membrane displacement at first resonance frequency of the system, while minimizing the membrane displacement at other frequencies. The feasible domain is defined by the six factors, namely $R_{m}$, $R_{b}$, $h_{c}$, $h_{g}$, $T_{m},$ and $t_{m}$. The ranges of the six factors used in the numerical experiments are presented in Table 3; these values were selected based on the process capabilities of our equipment to fabricate devices.

**Table 3:** List of experimental variables (factors) and their ranges.

Factors	Code	Range
Membrane Radius ($R_{m}$)	$x_{1}$	4–10 mm
Bottom Electrode Radius ($R_{b}$)	$x_{2}$	0.25–3 mm
Cavity Height ($h_{c}$)	$x_{3}$	1000–4000 μm
Air gap ($h_{g}$)	$x_{4}$	3–80 μm
Membrane Tension ($T_{m}$)	$x_{5}$	100–3000 N/m
Film Thickness ($t_{m}$)	$x_{6}$	8–25 μm

The variation domains of the six factors define a hypercube in six dimensions, and a second-order model was postulated to represent the evolution of the responses in this domain and optimize the acoustic resonant sensor. To estimate the coefficients of the model, a CCD was built with some additional points corresponding to a space-filling design to cover the entire domain. A total of 62 experiments were employed and listed in Table 4. These experiments were performed using numerical simulation, and for each experimental run, $C_{0}$, |˂*ξ_Se_*˃|*_fr1_*, $Q_{f}$, ∆C and |˂*ξ_Se_*˃|*_fr2_* were collected for further analysis and empirical model building. During this study, “nemrodW” statistical software [31] is used to develop experimental strategies and search for optimal settings.

**Table 4:** DOE Table for acoustic sensor study.

*N°Exp*	*R_m_*	*R_b_*	*h_c_*	*h_g_*	*T_m_*	*t_m_*	*N°Exp*	*R_m_*	*R_b_*	*h_c_*	*h_g_*	*T_m_*	*t_m_*
	mm	mm	µm	µm	N/m	µm		mm	mm	µm	µm	N/m	µm
1	4	0.25	1000	3	3000	8	32	10	3	4000	80	100	25
2	10	0.25	1000	3	100	8	33	10	0.25	1000	3	3000	25
3	4	3	1000	3	100	8	34	4	3	1000	3	3000	25
4	10	3	1000	3	3000	8	35	4	0.25	4000	3	3000	25
5	4	0.25	4000	3	100	8	36	10	3	4000	3	3000	25
6	10	0.25	4000	3	3000	8	37	4	0.25	1000	80	3000	25
7	4	3	4000	3	3000	8	38	10	3	1000	80	3000	25
8	10	3	4000	3	100	8	39	10	0.25	4000	80	3000	25
9	4	0.25	1000	80	100	8	40	4	3	4000	80	3000	25
10	10	0.25	1000	80	3000	8	41	10	1.625	2500	41.5	1550	16.5
11	4	3	1000	80	3000	8	42	7	0.25	2500	41.5	1550	16.5
12	10	3	1000	80	100	8	43	7	1.625	1000	41.5	1550	16.5
13	4	0.25	4000	80	3000	8	44	7	1.625	2500	3	1550	16.5
14	10	0.25	4000	80	100	8	45	7	1.625	2500	41.5	100	16.5
15	4	3	4000	80	100	8	46	7	1.625	2500	41.5	3000	16.5
16	10	3	4000	80	3000	8	47	5.9	1.322	2266	36.9	1407	15.8
17	4	1.625	2500	41.5	1550	8	48	8.1	1.322	2266	36.9	1407	15.8
18	10	1.625	2500	41.5	1550	8	49	7	2.231	2266	36.9	1407	15.8
19	7	0.25	2500	41.5	1550	8	50	7	1.625	3202	36.9	1407	15.8
20	7	3	2500	41.5	1550	8	51	7	1.625	2500	60.1	1407	15.8
21	7	1.625	1000	41.5	1550	8	52	7	1.625	2500	41.5	2265	15.8
22	7	1.625	4000	41.5	1550	8	53	7	1.625	2500	41.5	1550	20.8
23	7	1.625	2500	3	1550	8	54	4	0.5	1000	80	100	8
24	7	1.625	2500	80	1550	8	55	4	0.5	4000	80	3000	8
25	4	0.25	1000	3	100	25	56	10	0.5	4000	80	100	8
26	10	3	1000	3	100	25	57	7	0.5	2500	41.5	1550	8
27	10	0.25	4000	3	100	25	58	7	0.5	2500	80	1550	8
28	4	3	4000	3	100	25	59	4	0.5	4000	80	100	25
29	10	0.25	1000	80	100	25	60	4	0.5	1000	80	3000	25
30	4	3	1000	80	100	25	61	10	0.5	4000	80	3000	25
31	4	0.25	4000	80	100	25	62	7	0.5	2500	60.1	1407	15.8

## 5. Result and Discussion

### 5.1. Empirical Model Building and Analysis

To perform data analysis, the experimental data are first transformed into logarithmic scale to get symmetric distribution. The coefficients of the models are then estimated using common regression analysis techniques [27,32] to solve $X_{62\times 28}\beta_{28\times 1}=y_{62\times 1}$, where X indicates the matrix of factors and factor interactions, vector y is the experimental results for one response in logarithmic scale, and vector β is the unknown coefficients. Generally, β is estimated by resolving the linear system of equations, and can be expressed as $\beta ={\left(X^{T}X\right)}^{-1}X^{T}y$, where “T” and “−1” represent the transpose and inverse matrix, respectively. Once the coefficients are computed, the equation of the empirical model for each response is entirely defined. For simplicity only the most significant terms of the empirical models are mentioned in the Equations (20) to (24) below, although each second order polynomial response equation consists of 28 terms.

(20) $$Y_{C0}=0.29743+0.09489x_{1}+0.61423x_{2}-0.71485x_{4}-0.04947x_{1}^{2}-0.09846x_{2}^{2}+0.43464x_{4}^{2}-0.08399x_{1}x_{2}$$

(21) $$Y_{\left|\langle \xi_{Se}\rangle \right|fr1}=1.28557+0.22517x_{1}-0.87877x_{2}+0.25368x_{3}+0.95558x_{4}-0.24669x_{5}+0.11454x_{6}+0.51751x_{2}^{2}-0.46702x_{4}^{2}-0.29140x_{5}^{2}+0.14770x_{1}x_{2}+0.18454x_{1}x_{3}-0.06673x_{2}x_{3}-0.19040x_{2}x_{4}+0.09034x_{1}x_{5}+0.11223x_{2}x_{5}-0.06130x_{3}x_{5}+0.06152x_{4}x_{6}+0.13720x_{5}x_{6}$$

(22) $$Y_{Qf}=1.16077-0.45781x_{2}+0.09958x_{3}+0.12209x_{4}+0.09090x_{6}+0.75406x_{2}^{2}-0.31853x_{3}^{2}+0.24556x_{1}x_{2}+0.30512x_{1}x_{3}-0.19310x_{2}x_{3}-0.61056x_{2}x_{4}-0.16106x_{3}x_{4}-0.19291x_{1}x_{5}+0.15244x_{2}x_{5}+0.19565x_{3}x_{5}+0.36619x_{4}x_{5}+0.12421x_{2}x_{6}+0.17754x_{4}x_{6}$$

(23) $$Y_{∆C}=-0.11475+0.24282x_{1}+0.16241x_{2}+0.26101x_{3}-0.44159x_{4}-0.24636x_{5}+0.11822x_{6}+0.64695x_{4}^{2}+0.15244x_{1}x_{2}+0.19087x_{1}x_{3}-0.20920x_{2}x_{4}+0.09150x_{1}x_{5}+0.11199x_{2}x_{5}+0.14269x_{5}x_{6}$$

(24) $$Y_{\left|\langle \xi_{Se}\rangle \right|fr2\;}=0.43405+0.24954x_{1}-0.61280x_{2}+0.44395x_{4}-0.40514x_{5}-0.76316x_{4}^{2}+0.45812x_{6}^{2}-0.11145x_{2}x_{3}+0.21407x_{2}x_{4}+0.14111x_{2}x_{5}-0.15529x_{3}x_{5}-0.24556x_{4}x_{5}-0.24635x_{5}x_{6}$$

where *Y_C0_*, *Y_|˂ξ_se_˃|fr1_*, *Y_Qf_*, *Y_∆C_* and *Y_|˂ξ_se_˃|fr2_* represent the empirical models of the responses for $C_{0}$, |˂*ξ_Se_*˃|*_fr1_*, $Q_{f}$, ∆C and |˂*ξ_Se_*˃|*_fr2_*, respectively, and $x_{1}$, $x_{2}$, $x_{3}$, $x_{4}$, $x_{5}$, and $x_{6}$ are the coded values of $R_{m}$, $R_{b}$, $h_{c}$, *h_g_*, $T_{m}$ and $t_{m}$, respectively.

**Table 5:** Analysis of variance (ANOVA) table of estimated models.

Model	Source of Variation	Sum of Squares	Degrees of Freedom	Mean Square	Ratio	Sig.
***Y_C0_ = Log(C_0_)***	Regression	42.2128	27	1.5634	6205.1558	<0.01
	Residuals	0.0076	30	0.0003		
	Total	42.2204	57			
	R-Squared (R^2^)	1				
	Adj. R-Squared (R_a_^2^)	1				
***Y*_|˂*ξ*_*_Se_*_˃|*fr1*_*= Log(*|˂*ξ_Se_*˃|*_fr1_)***	Regression	99.2762	27	3.6769	112.9190	<0.01
	Residuals	1.0094	31	0.0326		
	Total	100.2856	58			
	R-Squared (R^2^)	0.990				
	Adj. R-Squared (R_a_^2^)	0.981				
***Y_Qf_ = Log(Q_f_)***	Regression	37.0305	27	1.3715	20.3269	<0.01
	Residuals	2.0242	30	0.0675		
	Total	39.0546	57			
	R-Squared (R^2^)	0.948				
	Adj. R-Squared (R_a_^2^)	0.902				
***Y_∆C_ = Log(∆C)***	Regression	24.8283	27	0.9196	14.0307	<0.01
	Residuals	2.0317	31	0.0655		
	Total	26.8600	58			
	R-Squared (R^2^)	0.924				
	Adj. R-Squared (R_a_^2^)	0.858				
***Y*_|˂*ξ*_*_Se_*_˃|*fr2*_*= Log(*|˂*ξ_Se_*˃|*_fr2_)***	Regression	43.6303	27	1.6159	18.1856	<0.01
	Residuals	2.7546	31	0.0889		
	Total	46.3849	58			
	R-Squared (R^2^)	0.941				
	Adj. R-Squared (R_a_^2^)	0.889				

To evaluate the significance of empirical models, analysis of variance (ANOVA) [33] is employed. The evaluated ANOVA of the model for logarithm of $C_{0}$, logarithm of |˂*ξ_Se_*˃|*_fr1_*, logarithm of $Q_{f}$, logarithm of ∆C, and logarithm of |˂*ξ_Se_*˃|*_fr2_* are summarized in Table 5, respectively. The column “Sig.” represents the *p*-values of the null hypothesis, which indicates the significance of the relation between factors and response, *i.e.*, the model significance. With the *p*-values being less than 0.01 in all five models, it can be concluded that the five response surface models are statistically significant with 99% confidence level. Furthermore, the goodness of the fit of the regression model is measured by R-Squared ($R^{2}$) and adjusted *R*-squared ($R_{a}^{2}$) values, which indicate the amount of variability in the response explained by the factors and range from 0 to 1. Therefore, the larger value is desirable. For all the models, $R^{2}$-values are closer to 1, thereby indicating that the regression line perfectly fits the data. On the other hand, $R_{a}^{2}$-value provides the predictive accuracy. From the table, it has been observed that the value of $R^{2}$ and $R_{a}^{2}$ are very close to each other, suggesting that the models for all responses are adequately reproducing the experimental data. This approach ensures the inclusion of only those variables that have a significant effect in the statistical model.

**Figure 7:**
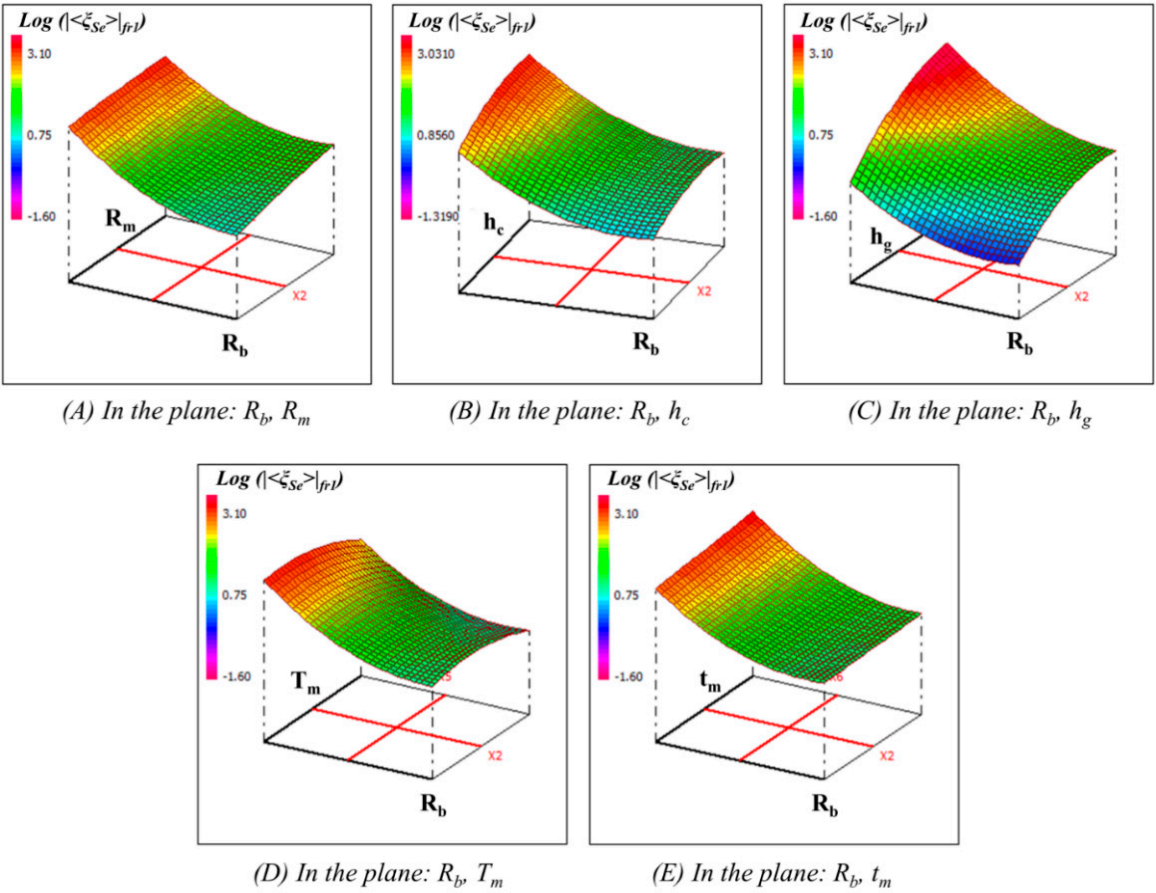
Response surface plots of logarithm of maximum membrane displacement at first resonance frequency (Log (|˂ξ_Se_˃|_fr1_)) in different planes with respect to other factors kept constants at the central values.

**Figure 8:**
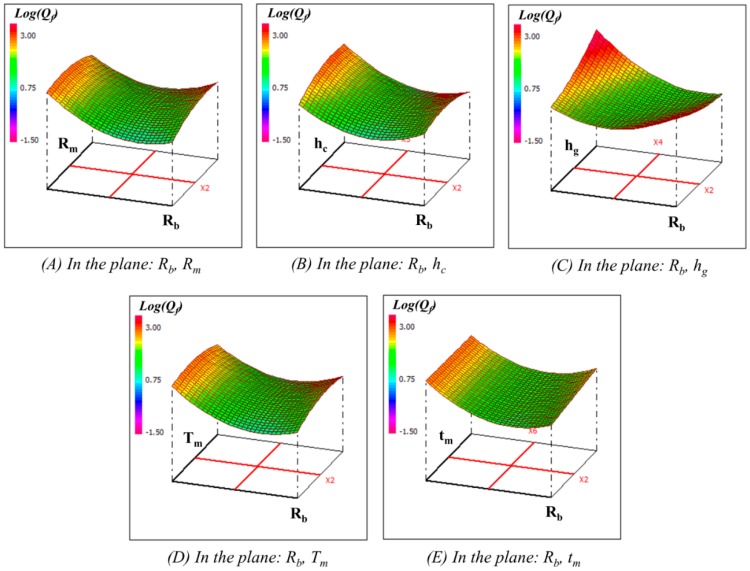
Response surface plots of logarithm of quality factor (Log(Q_f_)) in different planes with respect to other factors kept constant at the central values.

To further check the model behavior, response surface plots can provide a quick view to observe the maximum membrane displacement at first resonance frequency and the Q-factor for different values of factors and help to identify the type of interactions between these factors. Only two factors can be displayed on a plot while other factors are kept at constant levels at a central value. For example, the 3D graphical representations of the response surface of maximum membrane displacement at first resonance frequency and quality factor are illustrated in Figure 7 and Figure 8.

Figure 7A–E illustrate the interaction among different factors on the maximum membrane displacement at first resonance frequency for a fixed set of other factors at central values of their respective variation domain. It has been observed that all the six parameters, and some of the quadratic, as well as interaction between those parameters, have the strongest influence on the membrane displacement as presented by Equation (21). On the other hand, Figure 8A–E show the interaction of different factors on the quality factor of the membrane displacement at first resonance while other factors fixed at central levels. Observation reveals that the Q-factor has been influenced by linear terms $R_{b}$, $h_{c}$, $h_{g}$, $t_{m}$, quadratic terms $R_{b}^{2}$, $h_{c}^{2}$, and interaction terms $R_{m}R_{b}$, $R_{m}h_{c}$, $R_{m}T_{m}$, $R_{b}h_{c}$, $R_{b}h_{g}$, $R_{b}T_{m}$, $R_{b}t_{m}$, $h_{c}h_{g}$, $h_{c}T_{m}$, $h_{g}T_{m}$, $h_{g}t_{m}$ *etc.* The response surface plots also show the local maxima and minima of the responses in terms of different factors within their investigated ranges. As an example, membrane displacement of an acoustic resonator can be maximized by increasing the value of $R_{m}$, $h_{c}$, $h_{g}$ and $t_{m}$, and by reducing the value of $R_{b}$ and $T_{m}$ as illustrated by red color zone in Figure 7.

### 5.2. Optimization Process

As observed, the effects of factors are not only additive but also interactive. The presence of interaction effects makes it imperative that all the factors be optimized simultaneously to determine the best compromise and multi-criteria optimization is necessary. Desirability function approach is employed to achieve simultaneous optimization in our multi-response problems. In this approach, an objective function, also known as desirability function, is used to transform the existing values of the considered response in to a scale-free value called desirability. The desirability lies between 0 and 1 and it represents the closeness of a response to its ideal value.

**Figure 9:**
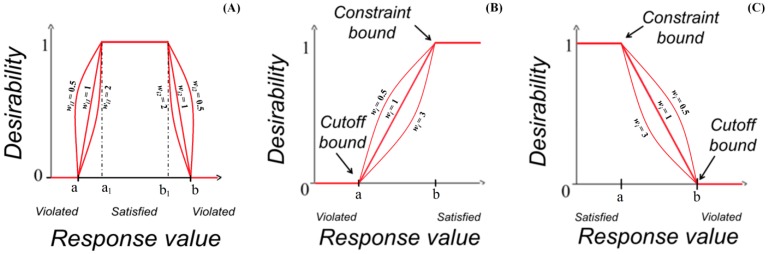
Schematic diagram of different desirability functions: (**A**) bilateral desirability function, (**B**) maximization and (**C**) minimization.

Multi-response optimization problem generally involves several processing steps after the models being fitted with the experimental data. Initially, the desirability index ($d_{i}$) was defined for each response, based on the part of desirability function as presented in Equations (25) to (27), for the cases of bilateral desirability function, maximization and minimization [11,15].

(25) $$d_{i}({\hat{Y}}_{i})=\{\begin{array}{c} 0\;\;\;\;\;\;\;\;\;\;\;\;\;\;if\;{\hat{Y}}_{i}<a\\ {\left(\frac{{\hat{Y}}_{i}-a}{a_{i}-a}\right)}^{w_{i1}}\;\;\;\;\;if\;a<{\hat{Y}}_{i}<a_{1}\\ \;\;\;\;\;\;\;\;\;1\;\;\;\;\;\;\;\;\;\;\;\;\;if\;a_{1}<{\hat{Y}}_{i}<b_{1}\\ {\left(\frac{b-{\hat{Y}}_{i}}{b-b_{1}}\right)}^{w_{i2}}\;\;\;\;\;if\;b_{1}<{\hat{Y}}_{i}<b\\ 0\;\;\;\;\;\;\;\;\;\;\;\;\;if\;{\hat{Y}}_{i}>b \end{array}$$

(26) $$d_{i}({\hat{Y}}_{i})=\{\begin{array}{c} 0\;\;\;\;\;\;\;\;\;\;\;\;\;if\;{\hat{Y}}_{i}<a\\ {\left(\frac{{\hat{Y}}_{i}-a}{b-a}\right)}^{w_{i}}\;\;\;\;\;if\;a<{\hat{Y}}_{i}<b\\ 1\;\;\;\;\;\;\;\;\;\;\;\;\;if\;{\hat{Y}}_{i}>b \end{array}$$

(27) $$d_{i}({\hat{Y}}_{i})=\{\begin{array}{c} 1\;\;\;\;\;\;\;\;\;\;\;\;\;\;if\;{\hat{Y}}_{i}<a\\ {\left(\frac{b-{\hat{Y}}_{i}}{b-a}\right)}^{w_{i}}\;\;\;\;if\;a<{\hat{Y}}_{i}<b\\ 0\;\;\;\;\;\;\;\;\;\;\;\;\;if\;{\hat{Y}}_{i}>b \end{array}$$

where “a” represents the lower tolerance limit, “b” represents the upper tolerance limit, and “$a_{1}$ and $b_{1}$” represent the target interval. The $w_{i}$, $w_{i1}$ and $w_{i2}$ in Equations (25) to (27) represent the considered weights. Shape of desirability functions are respectively illustrated in Figure 9.

Global desirability is then calculated by accumulating the “n” individual desirability values corresponding to the “n” studied responses, as follows,

(28) D=(∏i=1ndiwi)1∑wi

Here, D is the global desirability, $d_{i}$’ represents the respective individual desirability, and $\sum w_{i}\;$represents the total weight.

Thereafter, the optimum combination of levels of parameters is determined based on the highest global desirability value. Finally, the response of the sensor based on the optimum level of parameters is predicted and validated.

**Figure 10:**
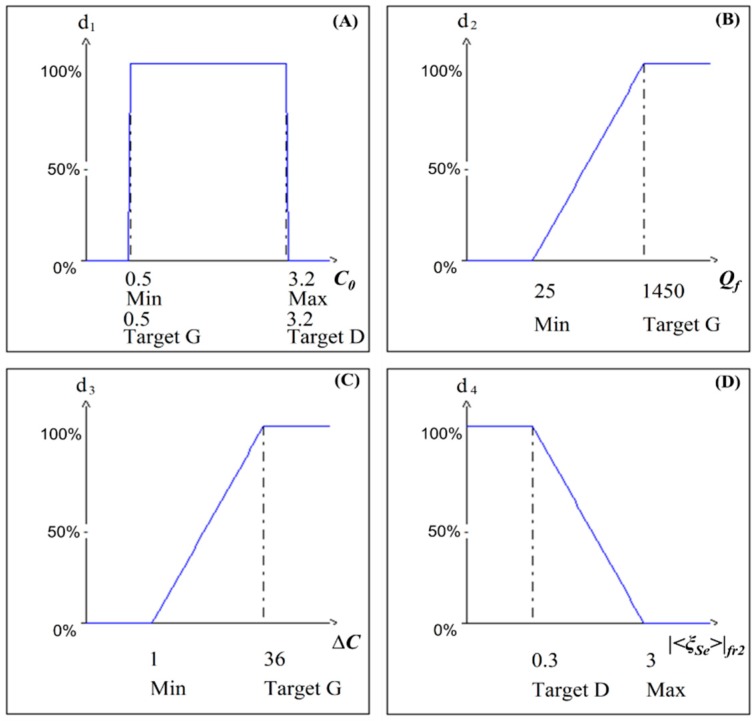
Desirability functions for multi-criteria optimization of acoustic resonant sensor (for (**A**) 0.5 ≤ $C_{0}$ ≤ 3.2 pF; (**B**) $Q_{f}$ ≥ 25; (**C**) ∆C ≥ 1fF; (**D**) |˂ξ_Se_˃|_fr2_ ≤ 3 nm).

In this study, the optimization is performed to obtain an acoustic resonant sensor, whereby the Q-factor and capacitance variation are maximized, while the value of static capacitance is held within the fixed value range and the membrane displacement at second resonance frequency is minimized to achieve better selectivity. Table 6 represents the list of optimization criteria and the desirability functions for responses that have been used during the optimization process. Figure 10A–D illustrate the desirability functions.

**Table 6:** Optimization criteria and desirability functions for the optimization of an acoustic resonant sensor.

Response (unit)	Partial Desirability Code	Functions	Weight (*w_i_*)	a	b	Predicted Response	Partial Desirability
C_0_ (pF)	*d_1_*	Bilateral	1	0.5	3.2	0.5	100%
Q_f_	*d_2_*	Maximization	1	25	1450	210	52.4%
∆C (fF)	*d_3_*	Maximization	1	1	36	1.72	15.1%
|˂*ξ_Se_*˃|*_fr2_*(nm)	*d_4_*	Minimization	1	0.03	3	1.12	21.3%

**Figure 11:**
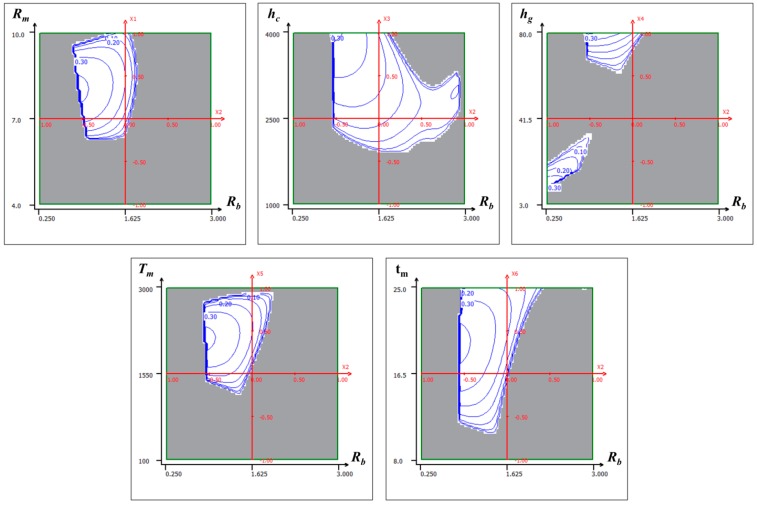
Optimum zone for acoustic resonant sensor with desired responses.

The solution found based on multi-criteria optimization is presented by response surface of global desirability with respect to different planes in Figure 11A–E, where white region represents the acceptable zone that satisfies all the criteria. Finally, the global desirability is evaluated based on which optimum level of parameters is decided to satisfy the desirability. The estimated set of optimized parameters based on multi-criteria optimization is listed in Table 7, which provides the global desirability of 36%.

**Table 7:** Set of optimized parameters based on multi-criteria desirability functions optimization.

Factor	Value
Membrane radius ($R_{m}$)	8.1 mm
Backplate radius ($R_{b}$)	0.871 mm
Cavity height ($h_{c}$)	3987 µm
Air gap ($h_{g}$)	80.0 µm
Membrane tension ($T_{m}$)	2158 N/m
Membrane thickness ($t_{m}$)	19.8 µm

### 5.3. Verification

**Figure 12:**
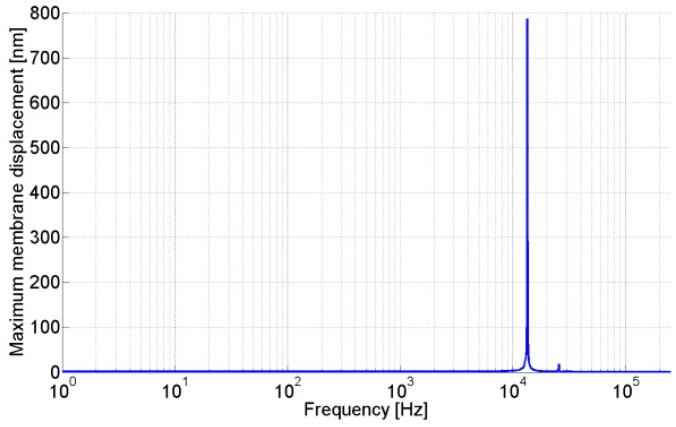
Maximum membrane displacement of the acoustic resonant sensor with set of optimum parameters.

Once the optimum set of parameters is determined, the numerical analysis has been performed to verify the responses of the acoustic sensor with optimum parameters. Figure 12 shows the maximum membrane displacement of the acoustic resonant sensor with respect to frequencies. It has been observed according to numerical analysis that the acoustic sensor with a set of optimum parameters provides good sensitivity and selectivity, with static capacitance ($C_{0}$) of 0.50 pF, capacitance variation (∆C) of 2.6 fF, and Q-factor ($Q_{f}$) of 522, alone with capacitance ratio ($∆C/C_{0}$) of 0.52% at first resonance frequency of the acoustic sensor for an incident acoustic pressure level of (or equal to) 80 dB_SPL_.

## 6. Conclusions

Numerical simulation and the DOE approach can be used to investigate the virtual prototyping of an acoustic sensor to understand the linear, quadratic, and interaction effects of different parameters on the outputs of the sensor. DOE helps to reduce the computation efforts in the acoustic resonant sensor optimization process since the empirical model is far less complex than the numerical simulation. RSM helps to develop empirical model for each response. It has been observed that the maximum membrane displacement at first resonance frequency and quality factor are influenced by several linear, quadratic, and interaction terms. Based on the empirical model, the region of the optimum set of parameters for an acoustic resonant sensor was obtained using multi-criteria optimization. During this work, global desirability of 36% was achieved. Cross-verification using numerical simulation shows that the capacitance of 0.50 pF, capacitance variation of 2.6 fF, and quality factor of 522 can be achieved. Hence, the optimum set of parameters satisfies the targeted output response of the acoustic resonator.
